# Stop Searching under the Streetlight! A Primer and Practical Guide to the Diagnosis of Joint Pain and Inflammation

**DOI:** 10.31138/mjr.33.3.291

**Published:** 2022-09-30

**Authors:** Elisha Krasin, Yoram Hemo, Ran Doron

**Affiliations:** 1. Maccabi Healthcare Services: Tel Aviv, Israel,; 2. Division of Orthopaedic Surgery, Tel Aviv Sourasky Medical Centre, affiliated with the Tel Aviv University Faculty of Medicine, Tel Aviv, Israel,; 3. Department of Paediatric Orthopaedics, Dana Children’s Hospital, affiliated with the Tel Aviv University Faculty of Medicine, Tel Aviv, Israel

**Keywords:** arthritis, arthralgia, differential diagnosis, arthrosis, tendinosis

## Abstract

It is impossible to achieve a diagnosis without a comprehensive list of possible diagnostic explanations for a certain symptom or syndrome. Joint complaints are very frequent in general practice, paediatrics, emergency medicine and naturally rheumatology and orthopaedics. The differential diagnosis of joint and surrounding tissue pain and inflammation is impressively challenging. Orthopaedic surgeons and rheumatologists deal with different aspects of this subject, while primary care physicians need to have a wider perspective that includes both orthopaedic and rheumatic disorders. The purpose of this paper is to provide a unified, comprehensive, clear and concise guide to this subject that will include both orthopaedic and rheumatic disorders and may serve the practitioner as basic reference for differential diagnosis. Short dictionary-style definitions would be given for each disorder in order to provide a ‘bird’s-eye view’, rather than an in-depth description of many diseases. Medical students, residents and primary care physicians are the primary target audience, but we believe that even the experienced orthopaedic surgeon or rheumatologist may benefit from a systematic and well-organised method.

## INTRODUCTION

While rheumatologists usually seek for symptoms and signs of inflammatory arthritides and various autoimmune disorders and orthopaedic surgeons seek for problems that can be treated surgically, patients unfortunately do not know whether they are “rheumatic” or “orthopaedic” and visit the doctor in order to receive a diagnosis and recommendations for treatment. This division is artificial; for example, a neglected scaphoid fracture may present years later, when the original trauma has long been forgotten, with pain and swelling of the wrist and radiological arthritis. On the other hand, inflammatory and rheumatic diseases can present with pain without signs of inflammation or deformity. The purpose of this paper is to provide a unified and practical guide to the diagnosis of joint and surrounding tissue pain and inflammation that includes both rheumatic and orthopaedic problems and to break the habit of “searching under the streetlight”.

Referred pain will not be discussed here in detail, but it is important to remember that joint pain is often referred from the spine or pathologies in the chest or the abdominal cavities. Simply saying, referred pain is characterised by absence of local signs and tenderness on palpation and motion of the affected joint. Chronic consequences of injury or overuse will be described here but generally we will not deal with acute traumatic injuries in this paper.

## PAIN AND INFLAMMATION OF A SINGLE JOINT

Rarely, monoarticular pain and inflammation can herald a systemic illness such as rheumatoid arthritis or rheumatic fever. In this section we will discuss local disorders that affect single joints with exclusion of systemic diseases that can specifically manifest as monoarthritis such as gout.

### Shoulder and acromioclavicular joint

Degenerative tears and tendinosis of the rotator cuff tendons inflict pain on motion and signs of impingement,^1^ though may also be asymptomatic. Cuff arthropathy is caused by massive tears of the rotator cuff tendons that leads to superior migration of the humeral head and abnormal wear on the head and glenoid.^2^ Osteoarthrosis of the glenohumeral joint is less frequent than osteoarthrosis of the ACJ (Acromioclavicular joint),^3^ and might be secondary to previous injury. Local tenderness over the ACJ and deformity are characteristic in ACJ arthrosis.^4^ Tears of the labral attachment of the biceps tendon (SLAP lesion) are more frequent in the dominant arm of high-performance overhead athletes,^5^ though may also appear without apparent history of injury or overuse. Labral tears are caused by direct trauma or chronic overuse. Instability of the glenohumeral joint or the ACJ due to previous injury, overuse or congenital hyperlaxity can present as shoulder pain.^6^ ‘Swimmer’s shoulder’ is an overuse shoulder injury in swimmers that combines impingement, hyperlaxity, labral injury and nerve entrapment.^7^ Synovial subacromial plica of the shoulder can cause impingement and shoulder pain.^8^ Previous shoulder dislocation may cause chronic pain due to a Hill-Sachs lesion, Bankart lesion or humeral avulsion of the glenohumeral ligament (HAGL).^9^

Inflammatory disorders of the shoulder and surrounding tissues can appear without local signs of inflammation due to the large mass of the deltoid muscle that obscures the joint. Bursitis and tendinitis around the shoulder are very common,^10^ tenderness on palpation of the affected bursa and tendon and pain on motion is typical. Ultrasonography may show swelling and accumulation of fluid in the bursae and thickening of tendons with surrounding fluid. Tendinitis of the long head of the biceps commonly occurs in weightlifters. Calcific tendinitis (HADD - Hydroxyapatite deposition disease) can present with sudden appearance of agonising shoulder pain. Characteristic tendon calcifications are seen on X-ray and ultrasonography.^11^ Frozen shoulder (adhesive capsulitis) causes pain and multidirectional limitation of motion.^12^ Post-capsulorrhaphy arthropathy can develop after previous shoulder stabilization surgery.^13^ ‘Coracoid syndrome’ is a local inflammation at the attachment of tendons and ligaments to the coracoid tip.^14^ Os acromiale can be occasionally responsible for shoulder pain.^15^ ‘Bench-presser’s shoulder’ is an overuse tendinopathy of the pectoralis minor muscle.^16^ ‘Milwaukee shoulder’ is a rapidly destructive arthritis of the shoulder, that appears at old age, with massive effusion, massive tears of the rotator cuff and depositions of calcium phosphate.^17^

Septic arthritis of the shoulder is less common than of other joints^18^ and will usually have an acute presentation with fever, chills and severe pain. The joint will be swollen and tender. Partially treated infections, infections with less virulent organisms and infections in immunosuppressed patients may have deceivingly less pronounced symptoms.^18^ Fungal infections^19^ and tuberculosis^18^ can have indolent course. Benign and malignant tumours of the proximal humerus are not rare.^20–22^ Pathological fractures on an existing asymptomatic bone cyst can produce sudden appearance of shoulder pain.^23^

Neuropathic arthropathy (Charcot arthropathy) may be found in patients with syringomyelia.^24^ Entrapment of the suprascapular nerve causes insidious onset of dull posterior shoulder pain.^25^ Quadrilateral space syndrome causes lateral shoulder pain and paraesthesia due to compression of the axillary nerve and the posterior humeral circumflex artery and appears especially in overhead athletes.^26^ Parsonage-Turner syndrome (brachial plexus neuritis) causes severe shoulder pain that radiates to the arm with sensory abnormalities and muscle weakness.^27^

Proximal humeral epiphysiolysis, “little league shoulder” is an overuse injury in overhead throwing adolescent athletes.^28^ Widening of the physis with adjacent sclerosis will be seen on X-ray. Avascular necrosis of the proximal humerus is usually post-traumatic; increasing insidious pain after a period of improvement is characteristic.^29^

### Sternoclavicular joint

Osteoarthrosis is the most common cause of localized pain and swelling.^30^ Post-traumatic pain may appear after dislocation. Pain and swelling could be a manifestation of SAPHO (Synovitis-acne-pustulosis-hyperostosisosteitis) syndrome, condensing osteitis and Friedrich’s disease (spontaneous necrosis of the medial end of the clavicle). Septic arthritis is not common and will cause local pain, chest pain, swelling, tenderness and fever.^30^ Tietze’s syndrome can also affect the sternoclavicular joint.^30^

### Elbow

Tendinopathies are the most common cause of elbow pain. Lateral epicondylitis (Tennis elbow) causes lateral pain, swelling and tenderness of the common extensor origin. Medial epicondylitis (Golfer’s elbow) is the inflammation of the common flexor-pronator origin. Tendinosis of the triceps tendon and the tendons of biceps and brachioradialis are less common.^31^ Olecranon bursitis is encountered frequently in clinical practice, mostly sterile but may be septic.^32^ Appearance of a soft and fluctuant tumour on the olecranon is typical. Primary osteoarthrosis of the elbow is rare and may be secondary to a previous intra-articular fracture.

Congenital dislocation of the radial head may present with pain and limitation of motion during childhood or adolescence.^33^ Plica in the radio-capitellar joint rarely can cause lateral elbow pain.^34^ ‘Little league elbow’ is an overuse injury in throwing adolescent athletes causing medial epicondylar pain.^35^ Lateral (osteochondrosis [Panner disease] or osteochondritis dissecans of the capitellum) and posterior (injury to the olecranon apophysis) injuries are also described.^35^ Radial tunnel syndrome (posterior inter-osseous nerve entrapment) may cause lateral elbow pain with possible motor weakness.^36^ Osteonecrosis of the elbow appears predominantly in steroid users^37^ and with other risk factors such as sickle-cell disease or Gaucher’s disease. In children can be post-traumatic. Other pathologies such as septic arthritis, synovial chondromatosis or a tumour naturally can affect the elbow as any other joint.

### Wrist and hand

Inflammation of tendons surrounding the wrist is extremely common and may be related to overuse. Swelling and tenderness over the affected tendon is usually seen, such as in De Quervain’s tenosynovitis.^38^ A ganglion cyst, that may be small and not palpable but painful, can be found on ultrasonography.^38^ Trigger finger can mimic monoarthritis of the metacarpophalangeal joint. Osteoarthrosis rarely affects the radio-carpal joint but is very frequent in the first trapezio-metacarpal (basilar arthritis), scapho-trapezoid-metacarpal, interphalangeal, second and third carpo-metacarpal joints (carpal boss) and the interphalangeal joints.^39^

Pseudogout (CPPD - Calcium Pyrophosphate Deposition) characteristically presents with acute inflammation of the wrist and effusion. Polarized light microscopy of the synovial fluid may show CPPD crystals.^40^ Haemochromatosis can cause clinically indistinguishable picture.^41^

Neglected scaphoid fractures with or without injury to the scapholunate ligament can present years later with arthritis of the wrist and carpal instability and collapse.^42^ Old intra-articular fractures and distal radio-ulnar joint injury can also cause development of secondary arthritis. Kienböck’s disease is a necrosis of the lunate of unknown aetiology that presents with chronic wrist pain.^43^ Avascular necrosis of other carpal bones may also be encountered but less frequently.^44,45^ Carpal instability is the disruption of the normal anatomic relationship of the carpus and radio-carpal joint usually due to trauma.^46^

Ulnar abutment syndrome is an overuse injury caused by load transfer between the distal ulnar pole and the carpus^47^ and may lead to tears of the triangular fibrocartilage complex. Degenerative or traumatic tears of the triangular fibrocartilage complex are the most common source for ulnar sided wrist pain.^48^ Intersection syndrome (oarsman’s wrist or squeaker’s wrist) is an overuse inflammation of the second extensor compartment.^38^ Other overuse syndromes are also described like hamatolunate impaction syndrome and dorsal wrist impingement syndromes.^38^ Gymnast’s wrist (distal radial physeal stress syndrome) is a common source for wrist pain in paediatric gymnasts.^49^ Madelung deformity can cause wrist pain and progressive deformity of the forearm bones.^50^ Negative and positive ulnar variances are associated with wrist pain and several of the above mentioned pathologies.^51^

Mauclaire or Dietrich disease^52^ is an osteonecrosis of the metacarpal heads that appears in adolescence and may lead to growth disturbance of the affected finger. Thiemann disease is osteonecrosis of the epiphyses of the phalanges that causes deformity of the fingers.^53^

### Hip and groin

Osteoarthrosis presents with pain on ambulation, morning pain and stiffness that gradually worsens during months and years. Sudden appearance of acute pain after physical effort or minor trauma is not rare. Though hip arthrosis is usually bilateral, one-sided presentation is frequent. Typical changes on X-ray with subchondral cysts are seen. Femoroacetabular impingement (FAI) is caused by a bony abnormality of the femoral head and neck that leads to early wear of the joint and labrum.^54^ Labral tears can appear even without injury or overuse and will cause anterior groin or lateral hip pain that worsens on effort.^55^ Osteochondritis dissecans of the hip is a rare entity.^56^

Tendinosis and strains of the large tendons that surround the hip joint are a frequent cause of pain. Localized tenderness over the gluteus medius tendon, the hamstrings or the adductors is suggestive to those diagnoses.^57^ Trochanteric bursitis is a common cause of tenderness over the trochanteric bursa. HADD around the hip is also a well-documented cause of ‘greater trochanter pain syndrome’^58^ or the involvement of the rectus femoris reflected head.^59^ Rectus femoris strains are not rare. In skeletally immature athletes, avulsion of the apophysis by the direct head will cause groin pain.^59^ Strains of the reflected head are common among adult athletes. Apophysitis of the lesser trochanter with avulsion can appear in boys involved in athletics.^59^ Internal snapping hip is caused by snapping of the psoas tendon and can be associated with iliopsoas bursitis.^59^ Core muscle injury also known as athletic pubalgia or sportsmen hernia is a complex injury to the pubic plate in athletes that includes stress reaction of bone, tendinosis and tears of the pubic plate (rectus abdominis, short adductors and adductor longus tendon), accumulation of fluid and development of cysts and arthrosis.^60^ External snapping hip syndrome is a painful subluxation of the junction between the iliotibial band and the gluteus maximus muscle over the greater trochanter.^59^ Morel-Lavallée lesion is a traumatic separation between the subcutaneous tissues and the fascia that can cause chronic inflammation and pain in the hip area.^59^ Synovial plica of the hip causes pain and a click with limitation of motion of the hip.^61^

Avascular necrosis of the hip can be idiopathic primary or secondary to injury, steroid use or alcoholism. Groin, lateral hip, or buttock pain are characteristic.^29^ Transient osteoporosis is a self-limited bone marrow oedema that may affect the hip in middle aged men or women during the third trimester of pregnancy or in the postpartum period ^62^. Stress fractures of the femoral neck are not rare.^63^ Septic hip presents with acute pain fever and leucocytosis. More indolent course is possible with less virulent bacteria, immunosuppression or in a partially treated infection.^18^ Psoas abscess can be also present. Tuberculosis of the hip is the second most prevalent site of extra pulmonary tuberculosis.^64^ The course is indolent and resembles osteoarthrosis. Familial Mediterranean fever (FMF) can present with monoarthritis of the hip.^65^

Perthes disease, slipped capital epiphysis and developmental dysplasia of the hip, all are possible causes for hip pain in the paediatric patient. Long term consequences of these disorders can present in adults as a degenerative joint disease.^66^ Transient synovitis of the hip is common in children younger than 10 and presents as sudden hip pain and limping.

### Sacroiliac joint

Osteoarthrosis of the SIJ (sacroiliac joint) is common.^67^ Acute pain can appear due to an inflammatory flare-up. Sacroiliac joint dysfunction, which characteristically has few abnormal imaging findings,^68^ can be responsible for local mostly mechanical pain. Bertolotti’s syndrome (lumbosacral transitional vertebra) can cause pain in the region of the SIJ.^69^ Stress fractures of the sacrum appear in osteoporotic patients.^70^ Septic arthritis of the SIJ will present with sudden appearance of pain, fever, chills and local signs of inflammation. Sacroiliac tuberculosis can present with prolonged pain with minimal, if at all signs of local sepsis.^71^ Local tumours and metastases^72^ may cause pain and should be excluded. Hyperparathyroidism can cause a brown tumour in the area of the SIJ.^73^ Osteonecrosis of the SIJ is extremely rare and may be related to Gaucher’s disease.^74^

### Knee

Osteoarthrosis of the knee joint is very common above the age of 40 years. Bilateral or unilateral pain that is getting worse on effort with morning pain and stiffness are the typical complaints. Inflammation of tendons and ligaments that surround the knee can be a part of osteoarthrosis or due to overuse. Pellegrini-Stieda syndrome is a calcification of the medial collateral ligament at the femoral attachment that may be post-traumatic or inflammatory. Tendinosis or tendinitis of the quad-riceps and patellar tendons are frequent with localized tenderness and often is related to overuse. Inflammation of other tendons and ligaments is not rare. Lateral pain and tenderness may be caused by the iliotibial band syndrome. Baker’s cyst appears below the medial head of the gastrocnemius and usually related to arthrosis or other intraarticular pathologies. In the paediatric population it may be congenital. Bursitis of the several large bursae of the knee, such as pes anserine bursa, will cause localized inflammation. Infrapatellar (Hoffa’s) or prefemoral fat pad impingement and inflammation appear after injury or chronic irritation and clinically mimic patellar and quadriceps tendinitis respectively.

Meniscal tears appear in younger persons mostly after injury and in older persons more often due to degeneration. Discoid meniscus is a congenital deformity, usually of the lateral meniscus that may be asymptomatic or may cause lateral knee pain and clicks. It is more prone to injury and tears and often bilateral. Meniscal cysts are found more often in the lateral meniscus, causing localized pain and swelling. Old anterior cruciate ligament injury, can present even years later with pain due to arthrosis. Chronic consequences after injury to other ligaments are less common. Injury to the upper tibio-fibular joint can lead to chronic lateral knee pain and instability.^75^ Arthrofibrosis of the knee presents as painful limitation of motion after knee injury or surgery.^76^

The aetiology of the patellofemoral pain syndrome is unknown and may be related to increased patellofemoral joint stress.^77^ It is a very common disorder; the characteristic presentation is anterior dull knee pain during and after effort in adolescents, mostly girls. Patellar instability is more common in females. The pathoanatomy is not completely understood, but may arise from dysplasia of the trochlear groove, patella alta, increased Q-angle and medial patellofemoral ligament insufficiency, that might be developmental or appear after injury.^78^ Patients may complain of persistent pain and instability and sometimes feeling a “pop” or a frank dislocation.

Osteochondritis dissecans of the knee presents as vague knee pain, often without injury, in adolescents and young adults.^79^ Secondary osteonecrosis of the knee appears in patients after organ transplantations, chemotherapy, Gaucher’s disease and those treated with high doses of steroids.^80^ Spontaneous osteonecrosis (SONK) or subchondral insufficiency fracture of the knee (SIF/SIFK) might be actually an insufficiency fracture or related to osteoarthrosis and appears in older patients. Postarthroscopic osteonecrosis develops after arthroscopic meniscectomy, chondroplasty, anterior cruciate ligament reconstruction and laser or radiofrequency surgery. Synovial plica of the knee, mostly medial, presents with pain localized medially to the patella with a click that aggravates on motion.^81^

FMF can present as knee monoarthritis.^65^ Pseudogout and acute gout cause an acutely inflamed knee.

Osgood-Schlatter disease is an apophysitis of the tibial tuberosity that is frequent in boys involved in athletics. Localized swelling, bony prominence and tenderness of the tibial tuberosity are pathognomonic.^82^ An avulsion injury is a rare complication of Osgood-Schlatter disease. Sinding-Larsen-Johansson disease is an apophysitis of the lower pole of the patella and is less common.^83^

In haemophilia and other coagulation disorders sudden appearance of knee swelling may be due to spontaneous hemarthrosis that may progress to chronic haemophilic arthropathy and contracture.^84^ Pigmented villonodular synovitis (PVNS) usually presents as painless swelling of the knee.^85^ Pain and functional disturbance appear later if untreated. Synovial chondromatosis mostly affects the knee. Pain that worsens with ambulation and swelling are typical presentations.^86^

Septic arthritis and subacute osteomyelitis (Brodie’s abscess) can affect the knee with symptoms similar to described in the previous paragraphs relating to other joints. Tuberculosis of the knee causes moderate swelling and pain with minimal calor. Local enlargement of the joint is characteristically seen.^64^ Intermittent hydrarthrosis is a rare form of arthritis that may affect the knee and cause transient recurrent joint effusions.^87^ Lipoma arborescens is a benign condition that is caused by sub-synovial villous proliferation of mature fat cells and presents as painless swelling and recurrent joint effusion of the knee. The synovial fluid is normal.^88^

Complex regional pain syndrome of the knee causes constant burning type pain and allodynia after injury that might be minor.^89^ Tabes dorsalis and other nerve disorders may cause neuropathic arthropathy of the knee.^24^

### Ankle and subtalar joint

Inflammation and tears of tendons and ligaments surrounding the ankle, whether acute or chronic, is very common. It can be related to overuse or previous injury but in many instances the reason is unknown. Achilles tendon inflammation with retrocalcaneal bursitis, peroneal tendinitis tibialis anterior and posterior and flexor hallucis longus inflammation are very common. Peroneal retinaculum injury with or without tendon subluxation and tears may be found following injury.^90^ Peroneus quartus is an accessory peroneal tendon that may cause stenosis and peroneal tendon tears. Os peroneum is mostly asymptomatic but can cause lateral ankle and foot pain.^90^ Previous lateral ankle ligaments or distal syndesmotic injury can lead to chronic lateral ankle pain and instability. Impingements of the ankle that may be anterolateral, medial or posterolateral, appear after injury or due to degeneration. Soft tissue mass and inflamed synovium can be demonstrated on imaging.^90^ Os trigonum syndrome appears in ballet dancers and footballers and is and manifested by posterior ankle pain on dorsiflexion.^91^ Sinus tarsi syndrome may follow injury, foot deformity or arthritis, causing pain and inflammation located in the sinus tarsi.^90^ Os subfibulare is an anatomic variant that may be symptomatic. Tarsal coalitions present with peroneal spastic flatfoot, recurrent sprains and ankle pain. Injury or neuroma of the sural nerve can cause lateral ankle pain and paraesthesia. Deep peroneal neuropathy and superficial peroneal neuropathy can present with chronic ankle pain.^90^ Charcot arthropathy appears in tabes dorsalis and common in other neurological injuries like diabetic neuropathy.

Osteoarthrosis of the ankle often appears after a previous fracture or ligamentous injury. Haemochromatosis typically affects the ankle joint.^92^ Non-union of a previously undetected fracture of the anterior process of the calcaneus may cause ankle and midfoot pain. Osteochondral lesions of the talar dome can cause lateral or anteromedial ankle pain. Stress fractures appear in running or marching athletes after drastic increase in activity or repeated excessive activity or in osteopenic and osteoporotic patients.^63^

Acute gout may affect the ankle joint causing sudden appearance of severe pain and signs of inflammation. Fever, leucocytosis and other systemic signs are possible. In chronic gout, tophi and periarticular erosion may be seen. FMF can present with ankle monoarthritis. Septic arthritis of the ankle presents with sudden appearance of pain, fever and signs of inflammation.

Intraosseous ganglion, giant cell tumour, osteoblastoma and other benign and malignant tumours of bone and soft tissues and metastases can appear in the ankle and should be considered in the differential diagnosis.^93^ ‘Shoe rim pseudotumor’ is a soft tissue mass caused by prolonged local pressure and appears in snowboarders and figure skaters.

Osteonecrosis of the talus and rarely other bones of the ankle can cause poorly localized ankle pain. It is a frequent complication of talar neck fractures and may also appear in chronic steroid use, Gaucher’s disease and sickle cell disease. Distal tibial epiphysitis is also described in adolescent athletes.^94^

### Midfoot

All the regular pathologies, such as tendinitis, arthrosis, ganglions, referred pain or neuropathic pain and stress fractures, can affect the midfoot joints.^95^ Tarsal boss is a bony prominence from the tarsometatarsal joint that is related to osteoarthrosis. Insertional tendinosis of the tibialis anterior or posterior will present with dorsal or plantar midfoot pain. Cuboidal fossa syndrome is peroneus longus tenosynovitis and tendinopathy in the fibro-osseous tunnel in the cuboidal fossa.^90^

Charcot arthropathy appears most often in patients with diabetic neuropathy and can lead to rapid destruction of the midfoot joints. Insufficiency fracture of the cuboid is not frequent and may cause lateral midfoot pain. Other midfoot Insufficiency fractures are quite rare. Previous traumatic injury to the Lisfranc or Shopart joints can cause midfoot arthrosis and pain. Accessory navicular bone or osteochondrosis (Kohler’s) may present with medial midfoot pain and tenderness over the navicular prominence in children. Iselin’s disease is an apophysitis of the base of the fifth metatarsal^96^ causing localized pain and swelling in adolescents. Müller-Weiss disease is an osteonecrosis of the tarsal navicular in adults.^97^ Gout typically can cause acute midfoot pain swelling and inflammation.

### Forefoot and toes

Osteoarthrosis frequently affects the toe joints causing pain and deformity. Hallux rigidus is arthrosis of the first metatarsophalangeal joint presenting with pain on ambulation, local tenderness, limitation of motion and palpable marginal joint osteophytes. Tendinitis of the toes is common and can mimic arthritis.^98^ Sesamoiditis can appear after injury, but more often is idiopathic or related to overuse. Localised pain and swelling are characteristic. Metatarsalgia is manifested by tenderness over the lesser toes’ metatarsal heads. Plantar plate lesions (tears and fibrosis) can cause metatarsal head pain.^98^ Freiberg’s infraction affects usually the second or third metatarsal head, causing localized swelling and pain in the acute phase that gradually improves. Morton’s neuroma presents with pain typically in between the third and fourth metatarsal heads. Trigger toe is quite rare and can mimic monoarthritis of the metatarsophalangeal joint. An inflamed bunion or bunionette presents with localized inflammation. Hammer and claw toes frequently present with joint pain and inflammation. Old injury such as turf-toe or intraarticular fractures of the toes can present with joint pain. The first metatarsophalangeal joint is the most common site for acute gout presenting with severe pain and inflammation.^99^ Thiemann disease is a rare form of osteonecrosis that affects the toe epiphyses.^53^

## SYSTEMIC DISORDERS THAT AFFECT JOINTS

Osteoarthritis causes multiple joint and spinal involvement with inflammation of periarticular tissues.^39^ Joint distribution is described in the previous paragraphs. Hereditary hemochromatosis causes a joint disease that resembles osteoarthritis but appears at an earlier age.^41^ Ankylosing spondylitis (AS) will cause axial pain often involving the sacroiliac joints and arthritis of hips and shoulders.^100^ Enteropathic arthritis is clinically similar to AS.^100^ Reactive arthritis appears one to four weeks after a genitourinary or gastrointestinal infection, and affects two to four joints of the lower extremities with enthesitis, spondylitis and asymmetrical sacroiliitis.^100^ Psoriatic rash is not mandatory for psoriatic arthritis to develop. Psoriatic nail changes are very common. The joint disease may take different patterns: asymmetrical oligoarticular arthritis, symmetrical polyarthritis, distal interphalangeal arthropathy, arthritis mutilans and spondylitis with or without sacroiliitis. Dactylitis (sausage fingers) is a characteristic finding. On X-ray, periostitis, enthesitis, productive changes, scattered and asymmetrical distribution, dactylitis and ankylosis are found.^101^

Rheumatoid arthritis affects mostly women and typically presents with polyarthritis characterised by morning stiffness, symmetrical involvement of the wrists, meta-carpophalangeal and proximal interphalangeal joints.^102^ The small joints of the feet may be similarly affected. Soft tissue swelling, joint deformities and extra articular manifestations are prevalent. Without treatment joint destruction is rapidly progressing; subluxations and flexion contractures develop up to ankyloses. On X-ray characteristic marginal erosions are seen.

In acute rheumatic fever migratory polyarthritis involving the large joints is observed in the majority of patients.^103^ Several variants of juvenile idiopathic arthritis are described; Systemic onset arthritis is characterized by systemic features: fever, rash, myalgia and serositis, with asymmetrical oligoarthritis. Enthesitis-related arthritis is a spondyloarthropathy with possible sacroiliitis. Oligoarticular, polyarticular and psoriatic variants are also known.^104^

Arthralgia with mild synovitis may be the presenting symptom of systemic lupus erythematosus (SLE). The arthritis of SLE is deforming in less than 10% and erosions are rare, hand and knee involvement is typical but any joint can be affected.^105^ Scleroderma patients frequently have joint manifestations.^106^ Arthritis can appear in the clinical presentation of several inflammatory myopathies.^107^ Sjögren’s syndrome joint manifestations are arthralgia, synovitis, morning stiffness and non-erosive arthritis.^108^ Mixed connective tissue disease is an overlap syndrome of scleroderma, SLE, polymyositis, and rheumatoid arthritis.

The vasculitides are a group of systemic disorders that are manifested by blood vessel inflammation and occlusion. All vasculitides are manifested by systemic signs like weight loss, fever, night sweats and arthralgia. Ischemia or infarction of specific organs or tissues varies between different syndromes. Henoch–Schönlein purpura is manifested in children by arthritis or arthralgia of feet and ankles and possibly knees, wrists, elbows, and hands in addition to cutaneous and renal manifestations. Any joint can be involved in gout with special predilection to the first metatarsophalangeal joint, the ankle and the knee. The disease typically starts with monoarthritis that transforms to polyarthritis, when chronic with multiple tophi.^99^ Pseudogout (Calcium pyrophosphate deposition disease) usually presents with knee or wrist monoarthritis or oligoarthritis that progress to chronic polyarthritis which can resemble osteoarthritis and often involve the second and third metacarpophalangeal joints.

Polymyalgia rheumatica will cause neck, symmetrical shoulder girdle, and hip pain, accompanied by low fever, fatigue, and malaise in adults older than 50. Remitting seronegative symmetrical synovitis with pitting oedema syndrome (RS3PE) is an arthritis characterized by symmetrical distal synovitis, pitting oedema of the hands and feet, negative rheumatoid factor, and good response to glucocorticoids, and may be associated with malignancy.^109^

Behçet’s disease characteristically presents with aphthous ulcers and genital ulcers accompanied by lower limb large joint arthritis or arthralgia, especially the knee. The arthritis is non-deforming and asymmetric and can be monoarticular, oligoarticular, or polyarticular. Symptoms appear in acute attacks and rarely become chronic.^110^ FMF presents with peritonitis, pleuritis or pericarditis, monoarthritis (hip, knee or ankle) and exertional leg pain.^65^ SAPHO syndrome can present with musculoskeletal pain, most often the anterior chest wall, thoracic and lumbar spine, in addition to the cutaneous manifestations. Sacroiliitis is frequent.^111^ Menopausal-associated arthralgia is a joint disease that is directly related to menopause, nevertheless after menopause, several autoimmune and degenerative disorders may worsen.^112^

The musculoskeletal manifestations of haemophilia are hemarthrosis leading to arthropathy, joint contractures and deformity.^84^ Sickle cell disease has many joint manifestations; avascular necrosis, secondary arthropathy, joint effusions and septic arthritis.^84^ The joint expressions of Thalassemia are arthritis and joint effusions.^84^ Scurvy causes swelling and tenderness of extremities and joints up to “pseudo-paralysis of scurvy”.^113^ Ochronosis can present with generalized bone pain and arthrosis.^114^

Many viral infections, such as measles, chickenpox, mumps**,** tick-borne encephalitis and haemorrhagic fevers present with arthralgia or even arthritis as a part of the clinical presentation. In acute infections, accompanied by high fever, chills and frequently a characteristic rash, the diagnosis of an infectious disease is quite obvious. When the infection is chronic like mononucleosis, hepatitis C or human parvovirus-B19, the situation is less clear and differentiation from rheumatic disorders is required. Coronavirus disease 2019 (Covid-19) patients reported myalgia and joint pain.^115^ Arthritis and arthralgia can be observed at any stage of human immunodeficiency virus infection.^116^

Systemic bacterial infections in their acute or chronic phases can present with arthralgia or arthritis. Diseases like brucellosis, gonorrhoea, Lyme disease, relapsing fever, epidemic typhus and Mediterranean spotted fever (Mediterranean boutonneuse fever) should be considered in the differential diagnosis when appropriate. Syphilitic disease of bone usually affects the diaphysis of long bones and is manifested by osteoperiostitis or gummatous inflammation. Osteolytic lesions, mimicking metastases are possible. In children, with inherited syphilis, inflammation of the epiphyses may also be observed. Charcot arthropathy can affect many joints.^117^ Skeletal tuberculosis progresses slowly, causing pain, swelling, tenderness and deformity of bones or joints (hips and knees most often). Back pain with deformity and various neurological abnormalities may appear (“Pott’s disease”). There is no calor over the infected area (“cold abscess”). Osteochondromatosis (Hereditary multiple osteochondromas) can cause multiple joint pain. Large proportion of patients diagnosed with hypertrophic osteoarthropathy (periostosis, clubbing, joint swelling and pain) are due to paraneoplastic syndrome.^118^

Gaucher’s disease causes severe pain of bones (“bone crisis”) and osteonecrosis of the femoral and humeral head. The typical “Erlenmeyer flask” deformity of the distal femur is seen on X-ray.^119^ Patients with hypermobility syndromes often suffer painful joint hypermobility.^120^

Paget’s disease of bone may cause joint pain and deformity.^121^ Musculoskeletal manifestations like arthritis and metabolic bone disease are common in celiac disease.^122^ Acute (Löfgren’s syndrome; triad of ankle arthritis that may spread to knees and other joints, hilar adenopathy and erythema nodosum) or chronic symmetrical, medium to large joint oligoarthritis are described in sarcoidosis.^123^ Amyloidosis arthropathy can mimic rheumatoid arthritis or polymyalgia rheumatica.^124^

A long list of drugs can cause arthralgia and arthritis: fluoroquinolones, statins, angiotensin converting enzyme inhibitors, antiepileptic drugs, antipsychotic drugs, bisphosphonates, cephalosporins, fibrates, immunosuppressive agents, iron chelating agents, nucleoside reverse transcriptase inhibitors, penicillins, proton pump inhibitors, isoretinoin and trivalent oral iron.^125^

## CONCLUSIONS AND DISCUSSION

Detailed history and physical examination will help us to find a way in this enormous subject. The first mission, when encountering a patient with joint complaints, is to find whether we are dealing with a systemic illness with articular manifestations, usually polyarticular, or a local disorder of a joint. It is not as simple as it seems, systemic disorders can start with monoarticular symptoms and local joint problems can attack several joints. Systemic signs such as fever, exhaustion, hyperhidrosis, weight loss, cutaneous signs (rash, tophi, purpura, psoriasis) and extra articular manifestations (renal, ocular, haematologic, pulmonary etc.), all point to a systemic illness. Elevation in acute phase reactants suggests infection or autoimmune/inflammatory disorder. History of injury is often a ‘red herring’ that distracts our attention from the actual diagnosis.

In systemic disorders, joint distribution can point to certain diagnoses. For example, affection of distal interphalangeal joints practically rules out rheumatoid arthritis but is characteristic for osteoarthritis and psoriatic arthritis. Plain X-rays of the hands are very helpful in differentiating different arthritides; marginal erosions are characteristic to rheumatoid arthritis, tophi with erosions typical to gout, periostitis and ankylosis to psoriatic arthritis. Tuberculosis of joints notoriously causes slowly progressive disease that resembles osteoarthrosis. Elevated acute phase reactants can assist in differentiation.

In local join disorders, careful examination of the joint may point to the structure that is responsible for pain. Swelling and tenderness of tendons, ligaments and bursae suggest inflammation or injury of those structures. Joint tenderness on palpation and motion with crepitus appear in various arthropathies. Provocation physical tests can suggest injury to menisci, labrum, and other anatomical structures. Plain X-rays may show soft tissue calcifications diagnostic to HADD, avascular necrosis, signs of previous injury, bone tumours and many other disorders. Ultrasonography becomes a standard for diagnosis of tendinopathies, strains and other soft tissue affections. The gold standard for diagnosis of joint disorders is magnetic resonance imaging (MRI) that demonstrates extremely well joint pathologies. MRI arthrography is preferred in examining the hip and shoulder joints and will improve detail in other joints. Gadolinium enhanced scan is recommended for tumours.

**Table 1.** Typical disorders of single joints that should be considered in differential diagnosis.

	**Shoulder and acromioclavicular joint**	**Sternoclavicular joint**	**Elbow**	**Wrist and hand**	**Hip**	**Sacroiliac joint**	**Knee**	**Ankle**	**Midfoot**	**Forefoot and toes**
**Tendinopathies, enthesopathies, impingement and overuse**	Degenerative tears and tendinosis of the rotator cuff tendons Cuff arthropathy Bicipital tendinitis Coracoid syndrome Bench-presser’s shoulder		Lateral epicondylitis Medial epicondylitis Tendinosis of the triceps, biceps, brachioradialis tendon	Inflammation of tendons surrounding the wrist (see text) Ganglion cyst Trigger finger Ulnar abutment syndrome Intersection syndrome (oarsman’s wrist or squeaker’ swrist) Hamato-lunate impaction syndrome and dorsal wrist impingement syndromes	Tendinosis and strains of the large tendons that surround the hip joint (see text) Strains of the rectus femoris reflected head Internal snapping hip Core muscle injury External snapping hip		Tendinosis and strains of the large tendons that surround the knee joint (see text) Pellegrini-Stieda syndrome Infrapatellar (Hoffa’s)or prefemoral fat pad impingement	Inflammation and tears of tendons and ligaments surrounding the ankle Peroneal retinaculum injury Peroneus quartus syndrome Os peroneum Previous lateral ankle ligaments or distal syndesmotic injury Impingements of the ankle Os trigonum syndrome Sinus tarsi syndrome Os subfibulare	Tendinitis and tendinosis (see text) Cuboidal fossa syndrome	Tendinitis (see text) Sesamoiditis Metatarsalgia Trigger toe Bunion or bunionette Hammer and claw toes Plantar plate lesions
**Bursitis**	Bursitis of the shoulder bursae		Olecranon bursitis		Trochanteric bursitis Psoas bursitis Morel-Lavallée lesion		Bursitis of the knee bursae Baker’s cyst	Bursitis of the ankle bursae		
**Arthrosis**	Osteoarthrosis primary or secondary – see text									
**Labral, meniscal and cartilaginous lesions, previous traumatic injury or surgery**	Tears of the labral attachment of the biceps tendon Labral tears Hill-Sachs lesion, Bankart lesion, Humeral avulsion of the glenohumeral ligament Post-capsulorrhaphy arthropathy Old intra-articular fractures	Old intra-articular fractures	Old intra-articularfractures	Tears of the triangular fibrocartilage complex Neglected scaphoid fractures with or without injury to the scapho-lunate ligament Old intra-articular fractures and distal radio-ulnar joint injury Carpal instability	Labral tears Old intra-articular fractures	Old intra-articular fractures	Meniscal tears Discoid meniscus Meniscal cysts Old Anterior cruciate ligament injury Injury to the upper Tibio-Fibular joint Arthrofibrosis of the knee Patellofemoral pain syndrome Osteochondritis dissecans of the knee Old intra-articular fractures	Previous fracture or ligamentous injury Non-union of a previously undetected fracture of the anterior process of the calcaneus	Old intra-articular fractures Previous traumatic injury to the Lisfranc or Shopart joints	Old intra-articular fractures Turf-toe
**Osteochondroses, epiphysitis and apophysitis, osteochondritis dissecans**	Proximal humeral epiphysiolysis, “little league shoulder”		“Little league elbow” Panner disease Osteochondritis dissecans of the capitellum Injury to the olecranon apophysis	Gymnast’s wrist (distal radial physeal stress syndrome)	Rectus femoris avulsion of the apophysis by the direct head Apophysitis of the lesser trochanter with avulsion Slipped capital epiphysis Osteochondritis dissecans of the hip		Osgood Schlatter Sinding Larsen Johansson	Osteochondral lesions of the talar dome Distal tibial epiphysitis	Kohler’s disease Iselin’s disease	
**Instability**	Instability of the glenohumeral joint or the ACJ “Swimmer’s shoulder”	Post dislocation instability	Post dislocation instability			Sacroiliac joint dysfunction	Patellar instability Post ligamentous injury instability	Ankle instability after ligamentous injury		
**Nerve entrapment or inflammation**	Neuropathic arthropathy (Charcot arthropathy) Entrapment of the suprascapular nerve Quadrilateral space syndrome Parsonage-Turner syndrome Referred pain	Referred pain	Radial tunnel syndrome (posterior interosseous nerve entrapment) Referred pain	Referred pain	Referred pain	Referred pain	Complex regional pain syndrome of the knee Neuropathic arthropathy Referred pain	Injury or neuroma of the sural nerve Deep peroneal neuropathy Superficial peroneal neuropathy Charcot arthropathy Referred pain	Charcot arthropathy Referred pain	Morton’sneuroma Referred pain
**Inflammatory disorders (hydroxyapatite deposition disease, calcium pyrophosphate deposition, gout etc.)**	Calcific tendinitis (Hydroxyapatite deposition disease) Frozen shoulder (adhesive capsulitis) Milwaukee shoulder	SAPHO syndrome Tietze’s syndrome Condensing osteitis		Pseudogout CPPD	HADD Familial Mediterranean fever Transient synovitis		Pseudogout Acute gout Familial Mediterranean fever Intermittent hydrarthrosis Lipoma arborescens	Acute gout FMF	Acute gout	Acute gout
**Septic arthritis and periarticular osteomyelitis**	Septic arthritis is possible at any joint Tuberculosis Epiphyseal subacute osteomyelitis (Brodie’s abscess)									
**Tumours and cysts, synovial proliferation**	Benign and malignant tumours, tumour like conditions Metastases Synovial chondromatosis Pigmented villonodular synovitis Ganglion cysts and intraosseous ganglion									
**Stress fractures**	Possible at any location after drastic increase in activity or repeated excessive activity									
**Pathological fractures**	Pathological fractures on an existing asymptomatic bone cyst or other bone weakening process									
**Avascular necrosis and Transient osteoporosis**	Avascular necrosis of the proximal humerus	Friedrich’s disease (spontaneous necrosis of the medial end of the clavicle)	Osteonecrosis of the elbow	Kienböck’s disease Avascular necrosis of other carpal bones Mauclaire or Dietrich disease Thiemann disease	Avascular necrosis of the hip Transient osteoporosis Perthes disease	Osteonecrosis of the sacroiliac joint	Osteonecrosis of the knee	Osteonecrosis of the talus and rarely other bones	Müller-Weiss disease	Freiberg’s infraction Thiemann disease
**Congenital and developmental deformities and dislocations, plicae**	Os acromiale Synovial subacromial plica		Congenital dislocation of the radial head Plica in the radio-capitellar joint	Madelung deformity Negative and positive ulnar variances	FAI Synovial plica of the hip Developmental dysplasia of the hip	Bertolotti’s syndrome (lumbosacral transitional vertebra)	Synovial plica of the knee	Tarsal coalitions	Accessory navicular bone	
**Bleeding (hemarthrosis)**	Possible in any joint in a patient with bleeding diathesis (hemophilia, anticoagulant treatment etc.)									
**Hyperpara thyroidism**	Brown tumour can develop at any joint									
**Pregnancy**	Especially affects pelvic joints									
**Systemic disease**	See text									

**Abbreviations:**HADD: Hydroxyapatite deposition disease; FMF: Familial Mediterranean fever; CPPD: Calcium pyrophosphate deposition; FAI: Femoroace-tabular impingement; SAPHO: Synovitis-acne-pustulosis-hyperostosis-osteitis syndrome.
